# Cation-dependent mannose-6-phosphate receptor expression and distribution are influenced by estradiol in MCF-7 breast cancer cells

**DOI:** 10.1371/journal.pone.0201844

**Published:** 2018-08-07

**Authors:** N. Bannoud, F. L. Carvelli, M. Troncoso, T. Sartor, L. M. Vargas-Roig, M. Sosa

**Affiliations:** 1 Instituto de Histología y Embriología (IHEM), Facultad de Ciencias Médicas, Universidad Nacional de Cuyo, CONICET, Mendoza, Argentina; 2 Instituto de Medicina y Biología Experimental de Cuyo (IMBECU), CONICET, Mendoza, Argentina; Roswell Park Cancer Institute, UNITED STATES

## Abstract

Cancer cells secrete procathepsin D, and its secretion is enhanced by estradiol. Although alterations in the pro-enzyme intracellular transport have been reported, the mechanism by which it is secreted remains poorly understood. In this work, we have studied the influence of estradiol on the expression and distribution of the cation-dependent mannose-6-phosphate receptor (CD-MPR), which would be a key molecule to ensure the proper localization of the enzyme to lysosomes in breast cancer cells. Immunoblotting studies demonstrated that the expression of CD-MPR is higher in MCF-7 cells, as compared to other breast cancer and non-tumorigenic cells. This expression correlated with high levels of cathepsin D (CatD) in these cells. By immunofluorescence, this receptor mostly co-localized with a Golgi marker in all cell types, exhibiting an additional peripheral labelling in MCF-7 cells. In addition, CD-MPR showed great differences regarding to cation-independent mannose-6-phosphate receptor. On the other hand, the treatment with estradiol induced an increase in CD-MPR and CatD expression and a re-distribution of both proteins towards the cell periphery. These effects were blocked by the anti-estrogen tamoxifen. Moreover, a re-distribution of CD-MPR to plasma membrane-enriched fractions, analyzed by gradient centrifugation, was observed after estradiol treatment. We conclude that, in hormone-responsive breast cancer cells, CD-MPR and CatD are distributed together, and that their expression and distribution are influenced by estradiol. These findings strongly support the involvement of the CD-MPR in the pro-enzyme transport in MCF-7 cells, suggesting the participation of this receptor in the procathepsin D secretion previously reported in breast cancer cells.

## Introduction

Cathepsin D (CatD) is a soluble aspartic protease that is overexpressed and secreted in high amounts by breast cancer cells [1, 2]. In primary breast carcinomas, the expression of this protein correlates with tumor progression and metastasis, therefore, it has been proposed as a marker of poor prognosis [3]. CatD is secreted as a pro-enzyme (proCatD), which can act as a mitogen on cancer and stromal cells, stimulating their pro-invasive and pro-metastatic capacities [4]. The CatD gene is controlled by a mixed promoter, which has both house-keeping and regulated gene features [5]. In this context, it has been well documented that, in hormone-responsive breast cancer cells, the transcription of CatD is induced by estradiol [6, 7]. In fact, the majority of cancer cell lines secrete over 50% of their proCatD production [2], being this secretion enhanced by estradiol [8].

In mammalian cells, under physiological conditions, most of CatD is confined to lysosomes and only between 5–10% of the precursor molecules are secreted [9]. CatD is synthesized in the rough endoplasmic reticulum as a pre-pro-enzyme and, after the removal of its signal peptide to generate proCatD, the molecule is glycosylated at two N-linked glycosylation sites to be transported to the Golgi apparatus. In the cis-Golgi network, proCatD is specifically modified by the addition of mannose 6-phosphate (M6P) residues to be targeted by mannose-6-phosphate receptors (MPRs) to lysosomes [10]. To be active, the pro-enzyme has to be processed to its mature form. This process begins in the late endosomes where the acidic pH favors the cleavage of proCatD (52 kDa) to render a single chain form (≈ 48 kDa), which reaches the lysosomes and is further processed to the mature two-chain form, i.e., CatD (≈ 33 and 14 kDa) [11].

Targeting of newly synthesized acid hydrolases by sortilin and LIMP-2 has also been proposed [12, 13]. Nonetheless, the M6P recognition pathway remains a key step in lysosomal targeting [14, 15]. Mammalian cells have two distinct MPRs: the 46 kDa cation-dependent MPR (CD-MPR) and the 300 kDa cation-independent MPR (CI-MPR). The biological significance of the existence of two MPRs with comparable function is still unclear. Both MPRs recognize M6P bearing ligands at the trans-Golgi network (TGN). Once recognized, these ligands are transported to an acidified pre-lysosomal compartment where the low pH causes the enzyme-receptor complex to dissociate. Consequently, the lysosomal enzymes are delivered to the lysosomes, while the receptors recycle back to the Golgi stacks [16]. Additionally, MPRs can also reach the cell surface [17] by several possible mechanisms, i.e., a missorting occurring at the TGN, through transport carriers derived from C5/6 stacks; and/or through recycling routes from either early [18] or late endosomes [19]. Although both receptors are present on the cell surface, only the CI-MPR can bind and internalize extracellular lysosomal enzymes and/or non-phosphorylated ligands such as insulin-like growth factor 2 (IGF2), meanwhile the CD-MPR has been proposed to mediate the secretion of hydrolase precursors [20].

The mechanism by which breast cancer cells secrete CatD is still poorly understood, although some hypotheses have been proposed. The increased expression of CatD together with a CI-MPR down regulation induced by estradiol [21], or a poor functionality of the receptor [22], would contribute to the enzyme secretion by a rapid receptor saturation mechanism. As for CD-MPR in tumor cells, little has been studied. Some authors have proposed that the contribution of the CD-MPR to the targeting of newly synthesized lysosomal enzymes becomes crucial only if the functionality of CI-MPR is deficient in non-tumorigenic cells. In that case, a fraction of the pro-enzymes that bind to the CD-MPR are secreted [20]. Given that intracellular transport alterations have been proposed to explain the exacerbated proCatD secretion by breast cancer cells and that CD-MPR has been scarcely studied in these cell types, in this work, we have studied the expression and distribution of CD-MPR in comparison with CI-MPR in a model of hormone-responsive breast ductal adenocarcinoma cell line, and attempted to correlate it with the expression and distribution of CatD, under basal conditions and under estradiol stimulation. The behaviour of the CD-MPR and its response to the hormone could be correlated with the exacerbated secretion of the pro-enzyme, a phenomenon that has been reported by other authors [1, 23].

## Materials and methods

### Antibodies and reagents

The goat anti-CatD antiserum was purchased from Santa Cruz Biotechnology (sc-6487 Dallas, TX, USA), and used 1:1000 in PBS for immunoblotting (IB) and 1:150 for immunofluorescence (IFI). The rabbit anti-CD-MPR antiserum was gently provided by Dr. Luzio (Cambridge University, UK), and used 1:250 for IB and 1:200 for IFI. The rabbit anti-CI-MPR antiserum was gently provided by Dr. Nancy Dahms (Medical College of Wisconsin, USA) and used 1: 500 for IB and 1:100 for IFI. The rabbit anti-LAMP1 antiserum (ab-24170) and mouse anti-β-tubulin monoclonal antibody (ab-56676) were obtained from Abcam (USA). The mouse anti-golgin97 monoclonal antibody was obtained from Santa Cruz Biotechnology (sc-73619). The HRP-conjugated anti-goat IgG antiserum was obtained from H&L (401515), the HRP-conjugated anti-rabbit IgG fraction was obtained from Sigma (A9169) and the HRP-conjugated anti-mouse IgG (whole molecule) was purchased from Sigma (A9044). Chemiluminescent reagents were from Pearce (Rockford, IL, USA).

### Cell cultures

Three breast cell lines were used in this study; the non-tumorigenic MCF-10A, and the tumorigenic, MCF-7 and MDA-MB-231 cell lines, obtained from the American Type Culture Collection (ATCC, Rockville, MD, USA). The MCF-10A is a human-derived mammary epithelial cell line which does not express estrogen receptor alpha (ERα), and whose characteristics are those of the normal breast epithelial cells [24]. The MCF-7 is a hormone-sensitive breast ductal adenocarcinoma-derived cell line expressing ERα. The MDA-MB-231 is also a breast ductal adenocarcinoma-derived cell line, but it presents a phenotype that is more mesenchymal than epithelial, and molecularly classified as triple negative (ER-/PR-/HER2-). All cell lines were used with 10–15 passages.

MCF-7 and MDA-MB-231 cell lines were cultured in DMEM Base (Sigma) supplemented with 10% charcolized fetal bovine serum (Internegocios), 2 mM L-glutamine (Sigma), 44 mM sodium bicarbonate, 1 mM sodium pyruvate, 5.6 mM glucose, 50 IU/50 μg/ml penicillin-streptomycin (Gibco) at 37°C under a 5% CO2 atmosphere.

The MCF-10A cell line was cultured in DMEM F12 HAM (Sigma), supplemented with 10% charcolized fetal bovine serum, 2 mM L-glutamine, 10 μg/ml insulin (Sigma), 10 ng/ml EGF (Sigma), 0.5 μg/ml hydrocortisone (Sigma), and 50 IU/50 μg/ml penicillin-streptomycin.

### Hormone treatments

MCF-7 and MCF-10A cells (60% confluence) were cultured as described above in T25 flasks with the corresponding culture media in the absence or in the presence of either 20 nM estradiol (Sigma) or 20 nM estradiol plus 2 μM tamoxifen (Sigma), for 12, 24 or 48 h. After incubation, cells were harvested after treatment with 0.1% trypsin (Gibco) for 5 min and processed for immunoblotting.

### Immunoblotting analysis

Cells were lysed with lysis buffer (PBS containing 1% Nonidet P-40 and 1mM PMSF), and homogenized by cellular disruption with a 0.5 mm needle followed by sonication. Proteins (40 μg) from the homogenates from each sample were resuspended in Laemmli’s buffer [25], and boiled for 5 min. For CI-MPR detection, homogenate proteins were resuspended in the Laemmli’s buffer without SDS and not boiled. Proteins were analyzed by electrophoresis in 6–10% polyacrylamide gels. The immunoblotting was carried out following the protocol of Romano et al. [26]. Briefly, after electrophoresis, proteins were electrotransferred onto nitrocellulose membranes (GE Healthcare, Amersham, Germany), for 4 h at 250 mA for detection of CI-MPR or for 1 h, at 250 mA for the other proteins under study. Membranes were blocked with 5% skimmed milk in buffer A (0.2% Tween 20 in PBS) for 1 h and incubated with the corresponding primary antibodies overnight at 4°C. After three washings with buffer A, membranes were incubated with the corresponding HRP-conjugated secondary antibodies. Specific bands were revealed by chemiluminescence and the signal was detected with a LAS 4000 imaging system (Fujifilm Lifescience, USA). Band intensities were quantified by densitometry using the Image J software (Image Processing and Analysis in Java; National Institutes of Health, Bethesda, MD, USA).

### Indirect immunofluorescence

Cells were grown on 1 cm diameter round coverslips seated at the bottom of culture wells under the conditions described above. Once 50% confluence was reached, cells were washed once with PBS and fixed with 3.7% paraformaldehyde for 20 min. Subsequently, cells were permeabilized with 0.1% saponin for 15 min, washed three times with PBS and blocked with 5% horse serum for 30 min. Afterwards, cells were incubated with primary antibodies, at the dilution indicated above, overnight at 4°C, then washed three times with PBS, and incubated with the corresponding fluorochrome-conjugated secondary antibodies diluted in PBS-horse serum for 90 min. Cell nuclei were stained with Hoescht, and coverslips were mounted on slides with Mowiol mounting solution. Samples were analysed with an Olympus FV 1000 confocal microscope and images were acquired using the FV 10-ASW 1.7 software (Olympus, Japan).

### Quantitative co-localization analysis

The co-localization analysis was carried out with the JACoP plugin of the Image J software (NIH [http://rsb.info.nih.gov/ij/plugins/track/jacop.html]). Pearson and Mander correlation coefficients (PCC and MCC, respectively) were calculated. For PCC, the dependency of pixels in dual-channel images (green/red channel for detection of CD-MPR/CatD; LAMP-1 /CatD and CD-MPR/Golgin) was assessed by plotting the pixel grey values of two images against each other. These values were displayed in a pixel-distribution diagram (scatter plot), and a linear equation describing the relationship between the intensities of the two images was determined by linear regression. A cross-correlation function (CCF) was obtained by plotting the corresponding PCC for each pixel shift (δx) of the green image in the x direction relative to the red image, or viceversa. The PCC value varied from 1 to -1, where values of 1 denote complete correlation, while values of -1 suggest a negative correlation. Among the MCC coefficients, the MCC-M1 and MCC-M2 were useful to describe the proportion of each protein that co-localized with the other, since this coefficient is independent of the fluorophore fluorescence intensity. The MCC values varied from 0 to 1, indicating no co-localization or complete co-localization, respectively.

### Discontinuous sucrose gradients

Subcellular fractions from MCF-7 cells subjected or not to hormone treatment, were obtained in discontinuous sucrose gradients, according to other authors [27]. Briefly, MCF-7 cells were harvested and homogenized with a teflon Dounce tissue homogenizer in buffer B (10 mM Tris-HCl pH 7.4, containing 0.25 M sucrose, 1 mM EDTA, and 0.02% PMSF). Homogenates were centrifuged at 3,000 g for 10 min and the resulting supernatants were then centrifuged at 30,000 g for 20 min. Pellets were then resuspended in buffer B, and the protein concentration was determined according to Lowry [28]. Each membrane sample was loaded on top of a 20–50% (w/w) discontinuous sucrose gradient (prepared in buffer C (10 mM Tris-HCl (pH 7.4), containing 1 mM EDTA, 0.02% PMSF) and centrifuged at 100,000 g for 60 min at 4°C. Fractions of 1 mL were collected from the bottom and weighed to estimate the fraction density (W/V). Subsequently, 2 ml of buffer C were added to each fraction, and centrifuged at 30,000 g for 30 min at 4°C to remove sucrose. Final pellets were processed for immunoblotting.

### Statistics

Data were analysed by the Tukey-Kramer multiple comparisons test. The level of significance was set at *p*≤0.05. At least three independent experiments were performed in each case.

## Results

### Tumorigenic and non-tumorigenic breast cell lines express different levels of CatD, CD-MPR and CI-MPR

**Fig 1:**
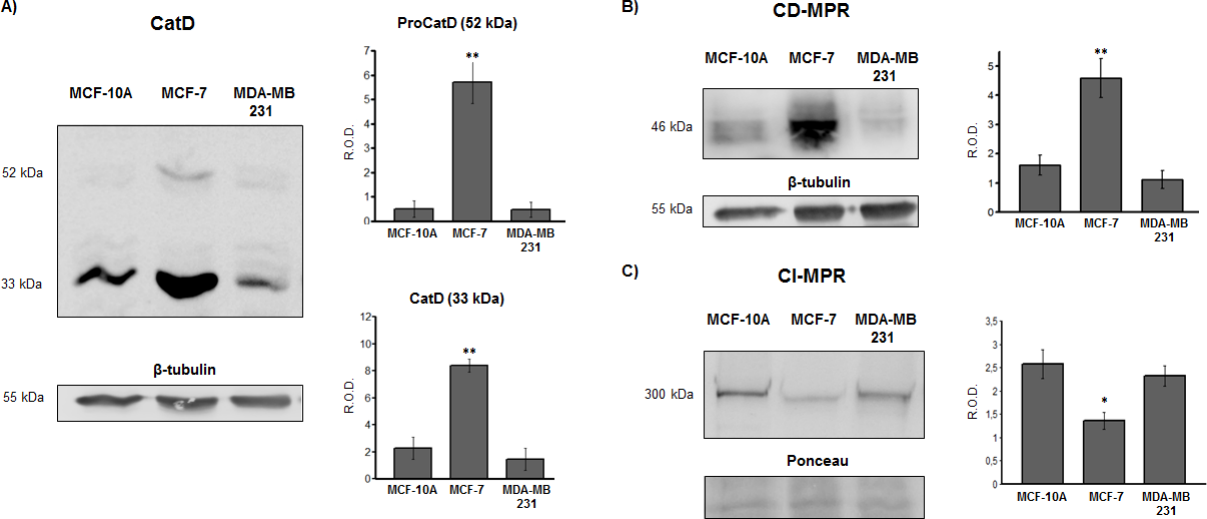
Expression of CatD, CD-MPR and CI-MPR in non-tumorigenic and tumorigenic human breast cell lines. MCF-10A, MCF-7 and MDA-MB-231 cell lysates were analyzed by immunoblotting. Representative immunoblottings of CatD (A), CD-MPR (B) and CI-MPR (C) with their band intensity quantitation. ProCatD (52 kDa, upper panel) and CatD (33 kDa, lower panel) were quantified separately. Bars represent the means of relative optical density (R.O.D.) ± SEM from four independent experiments for each protein. (**) and (*) significant differences (p <0.01 and p<0.05, respectively). Detection of β-tubulin and Ponceau red staining were used as loading control.

The expression levels of the proteins under study were evaluated by immunoblotting. As observed in Fig 1A, the mature form of CatD (33 kDa) is highly expressed in MCF-7 tumorigenic cells and, at a lesser extent in non-tumorigenic (MCF-10A) and in tumorigenic triple negative MDA-MB-231 cells. Moreover, the levels of the immature form of the enzyme (52 kDa) were also significantly higher in MCF-7 cells, indicating that the total expression of the enzyme is increased in these cells. A similar trend was observed for the CD-MPR, since the expression of this receptor was higher in the MCF-7 cells than in the MCF-10A or MDA-MB-231 cells (Fig 1B). In contrast, the CI-MPR expression levels were significantly higher in MCF-10A and MDA-MB-231 than in MCF-7 cells (Fig 1C).

### The subcellular distributions of CatD and CD-MPR differ between tumorigenic and non-tumorigenic breast cell lines

**Fig 2:**
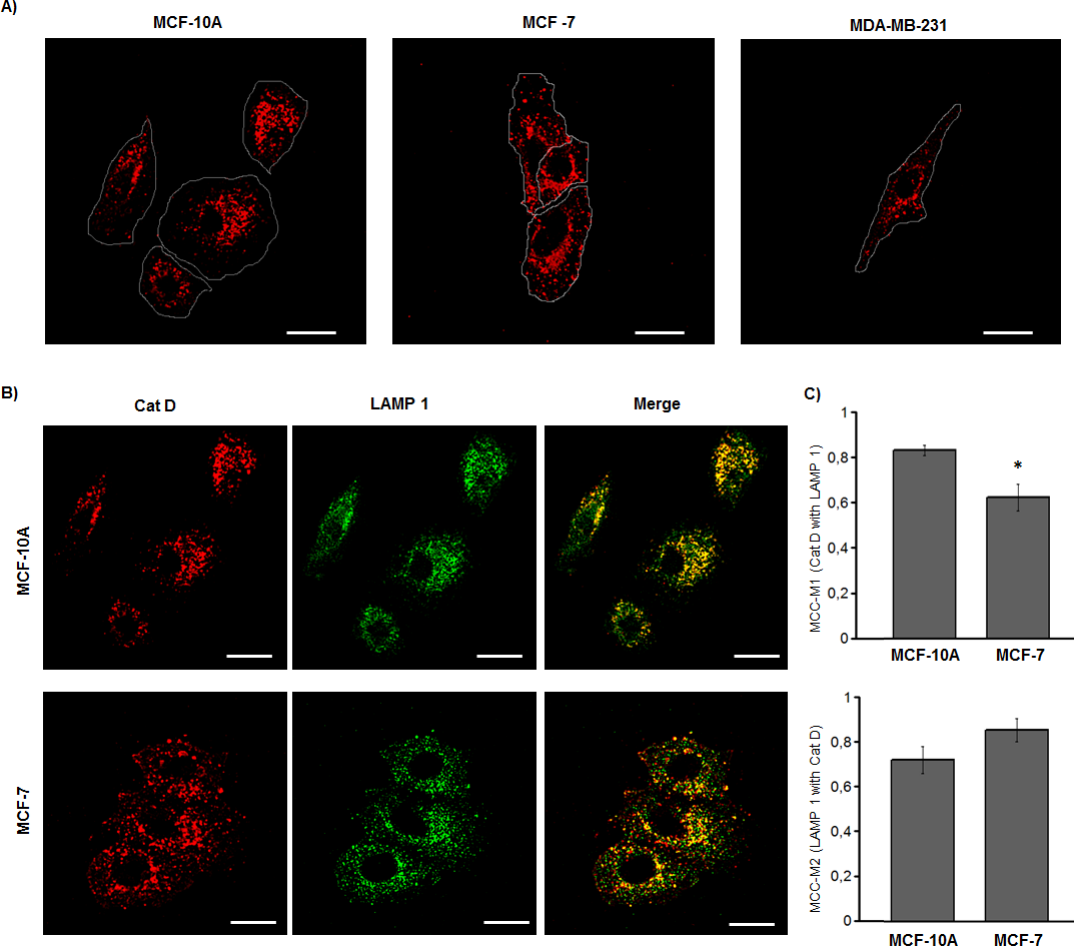
Cathepsin D distribution in non-tumorigenic and tumorigenic human breast cell lines. (A) Representative CatD location in MCF-10A, MCF-7 and MDA-MB-231 cell lines. (B) Co-localization of CatD and LAMP 1 in the MCF-10A and MCF-7 breast cell lines. (C) Quantitation of co-localization of CatD and LAMP1 (MCC-M1); and LAMP1 and CatD (MCC-M2) in MCF-10A and MCF-7 cell lines. Values are expressed as the means of Manders co-localization coefficients 1 and 2 (MCC-M1 and MCC-M2, respectively) ± SEM. (*) significant difference from MCF-10A (p <0.05). Scale bar = 20μm.

By IFI, a perinuclear location and a granular cytoplasmic distribution of CatD were observed in the three breast cell lines. However, an additional peripheral punctuated distribution of the enzyme, neighbouring the plasma membrane, was also observed in MCF-7 and MDA-MB-231 cell lines, but not in MCF-10A cells (Fig 2A, arrows). From these observations we evaluated the degree of co-localization of the enzyme with the lysosomal associated membrane protein 1 (LAMP1) and compared it between MCF-7 and MCF-10A cells, since among these cells the major difference in CatD distribution was observed. Although CatD showed partial co-localization with LAMP1 in MCF-7 and MCF-10A cell lines (Fig 2B), this co-localization was significantly lower in MCF-7 cells (Fig 2C), indicating the occurrence of higher location of the enzyme in non-lysosomal/endosomal compartments, when compared to MCF-10A cells.

**Fig 3:**
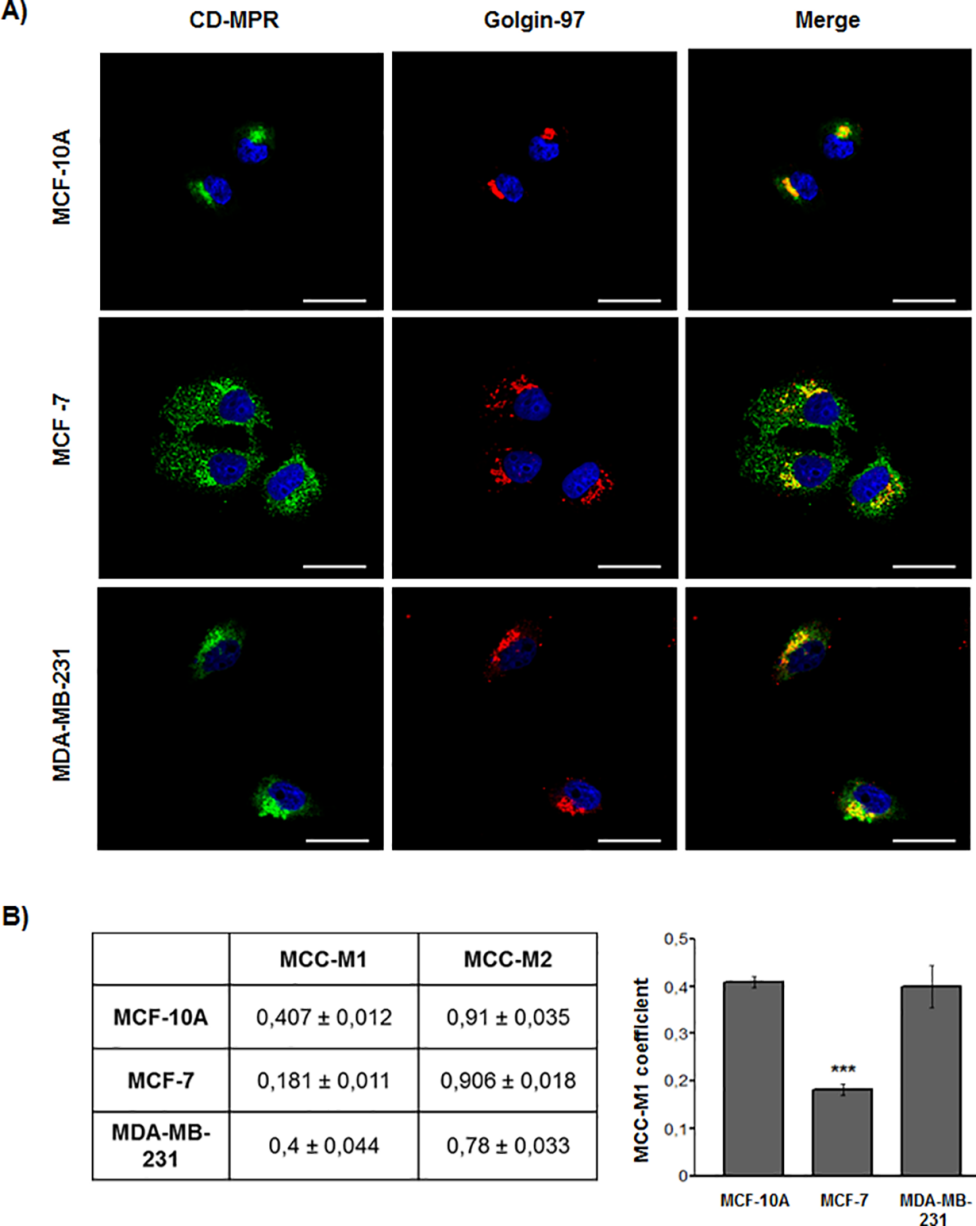
CD-MPR distribution in non-tumorigenic and tumorigenic human breast cell lines. (A) Representative immunofluorescence staining of CD-MPR and golgin-97 in MCF-10A, MCF-7 and MDA-MB-231 cell lines. Golgin-97 was used as a TGN marker. (B) Quantification of co-localization of CD-MPR and golgin-97 (MCC-M1); and golgin-97 and CD-MPR (MCC-M2). Values are expressed as the means of Manders co-localization coefficients 1 and 2 ± SEM. (***) significant differences from the other cell lines (p <0.001). Scale bar = 25 μm.

On the other hand, the CD-MPR localization was found to be mostly perinuclear in the three cell lines (Fig 3A). Such location would correspond to the Golgi apparatus, as evidenced by the simultaneous reactivity with golgin-97. However, in MCF-7 cells, an additional punctuate CD-MPR labelling was observed dispersed in the cytoplasm. Such reactivity did not co-localize with golgin-97 (Fig 3B). It is worth mentioning that by IFI, an apparent higher CD-MPR signal was observed in MCF-7 cells. The latter finding is in line with the higher expression observed by immunoblotting (Fig 1B).

### CD-MPR and CI-MPR display a differential distribution in MCF-7 cells

**Fig 4:**
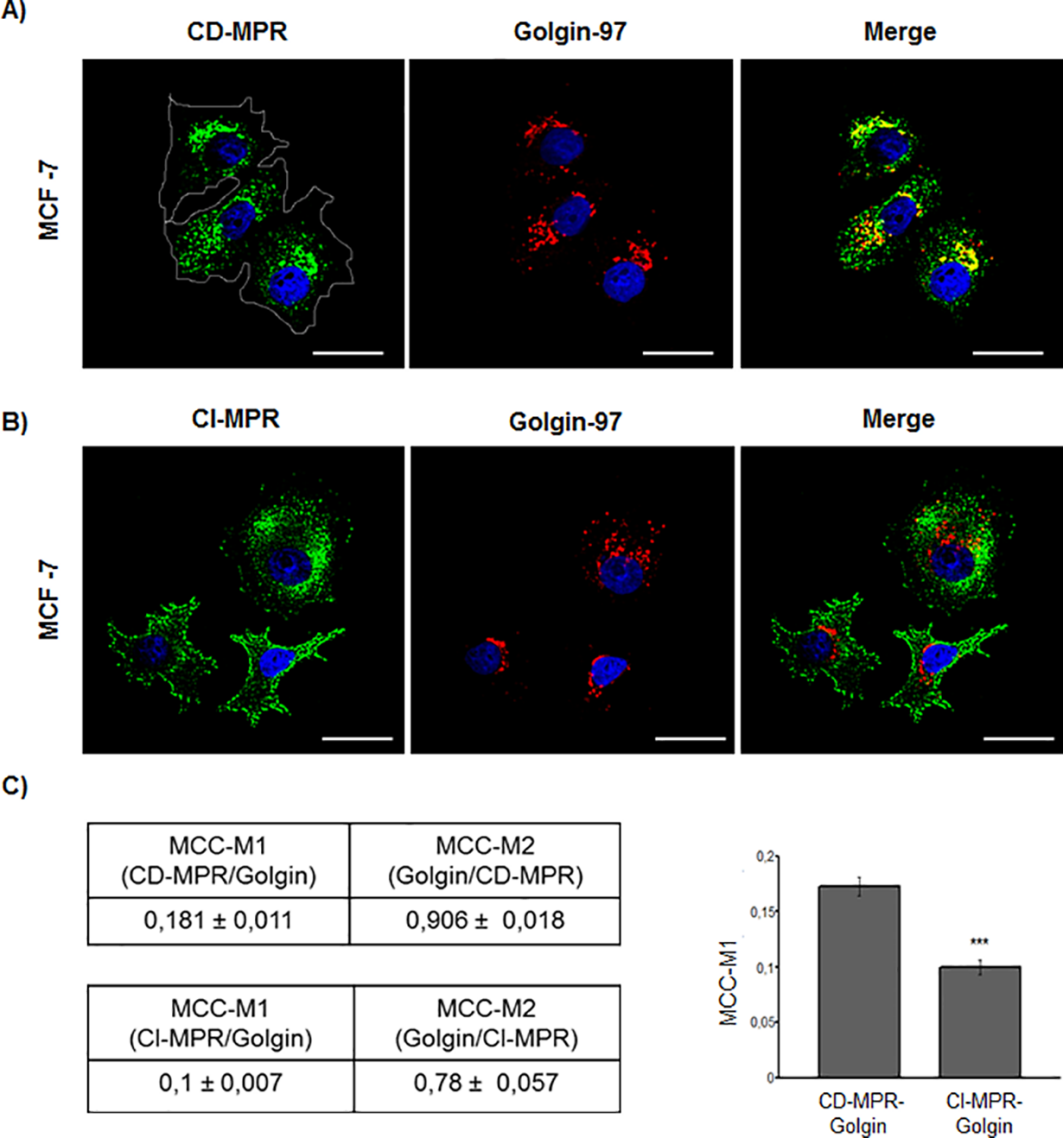
CD-MPR and CI-MPR co-localization analysis in the MCF-7 tumorigenic cell line. Representative immunofluorescence staining of (A) CD-MPR and golgin-97 and (B) CI-MPR and golgin-97 in MCF-7 cells. (C) Quantitative co-localization analysis showing the proportion of CD-MPR that overlaps with golgin-97 (Manders coefficient 1 –MCC-M1) and the proportion of golgin-97 that overlaps with CD-MPR (MCC-M2). A similar analysis was done for the CI-MPR. Values are expressed as the means of Manders co-localization coefficient 1 ± SEM. (***) significant difference (p <0.001). Scale bar = 25 μm.

The CD-MPR and CI-MPR co-exist in most mammalian cells; however, the biological significance for such co-existence is still unknown. Although the CI-MPR has already been studied in MCF-7 cells, no comparative studies have been performed between both MPRs in this cell line. Therefore, we analysed the distribution of the CI-MPR and compared it with that of CD-MPR in the MCF-7 cell line. As observed in Fig 4B, CI-MPR showed a dispersed cytoplasmic distribution with a signal appearing in the cell periphery, suggesting its presence in the plasma membrane, in contrast with the perinuclear CD-MPR distribution (Figs 3A and 4A). Moreover, only 10% of CI-MPR co-localized with golgin-97, indicating a major location outside the Golgi stacks for this receptor.

**Fig 5:**
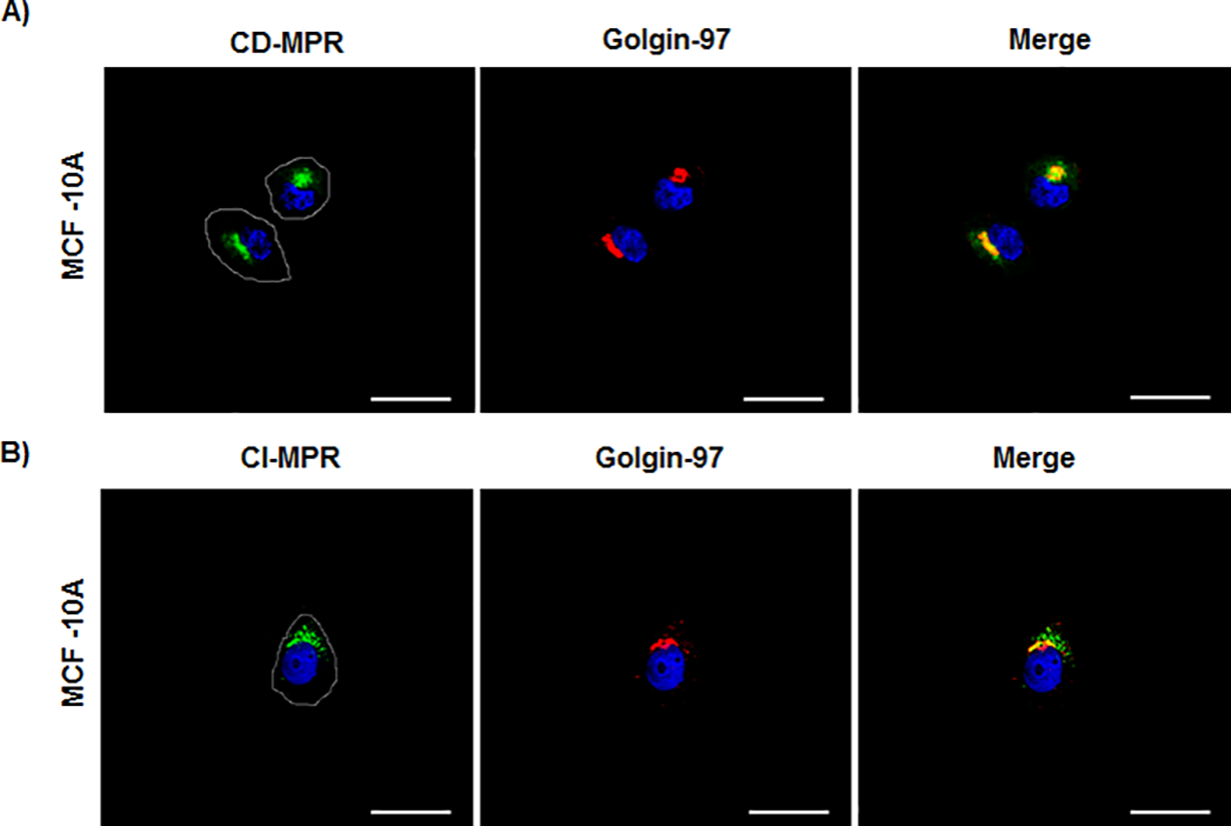
Immunofluorescence of CD-MPR and CI-MPR in the non-tumorigenic MCF-10A cell line and co-localization with golgin-97. (A) Representative immunofluorescence staining of CD-MPR and golgin-97 and (B) CI-MPR and golgin-97 in MCF-10A cells. Cell borders are delimited by a white line. Scale bar = 25 μm.

On the other hand, unlike MCF-7 cells, in the non-tumorigenic MCF-10A cells, both CD-MPR and CI-MPR showed a more concentrated perinuclear localization with high co-localization with golgin-97 (Fig 5).

### Estradiol regulates the expression and distribution of CatD and CD-MPR in MCF-7 cells

Taking into account that CatD and CD-MPR are highly expressed in the tumorigenic MCF-7 cells, and that an estrogen response element is present in the CatD gene, we evaluated the effect of 17-β-estradiol on CatD and CD-MPR expression and distribution on this estrogen-responsive cell line.

**Fig 6:**
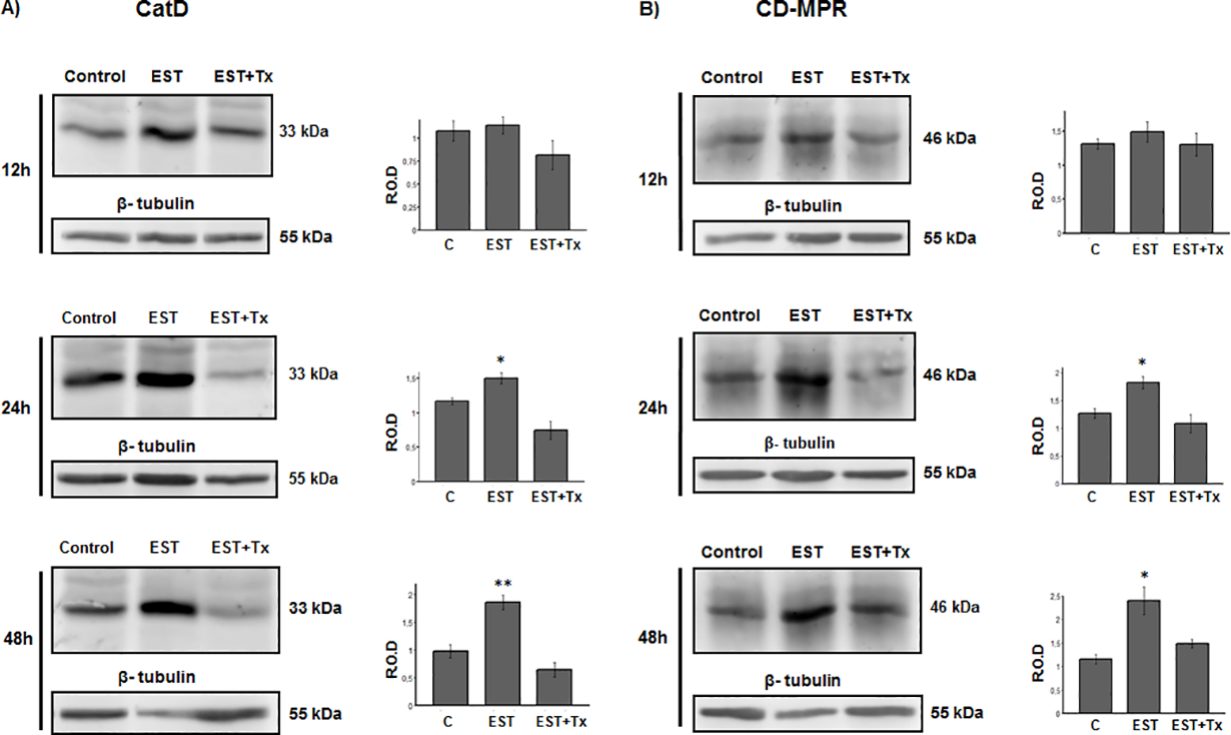
Effect of 17-β-estradiol on CatD and CD-MPR expression in MCF-7 cells. Cells were cultured for 12, 24 or 48 h in the absence (control) or in the presence of 20 nM estradiol (EST) or 20 nM EST with 2 μM tamoxifen (EST+Tx). Representative immunoblots of CatD (A) and CD-MPR (B) from cell lysates at the different timepoints and the corresponding band intensity quantitation by densitometry (bars graphics). Values are expressed as the means of relative optical density (R.O.D.) from three independent experiments ± SEM. (*) significant difference from the other conditions (p <0.05). Detection of β-tubulin was used as loading control.

**Fig 7:**
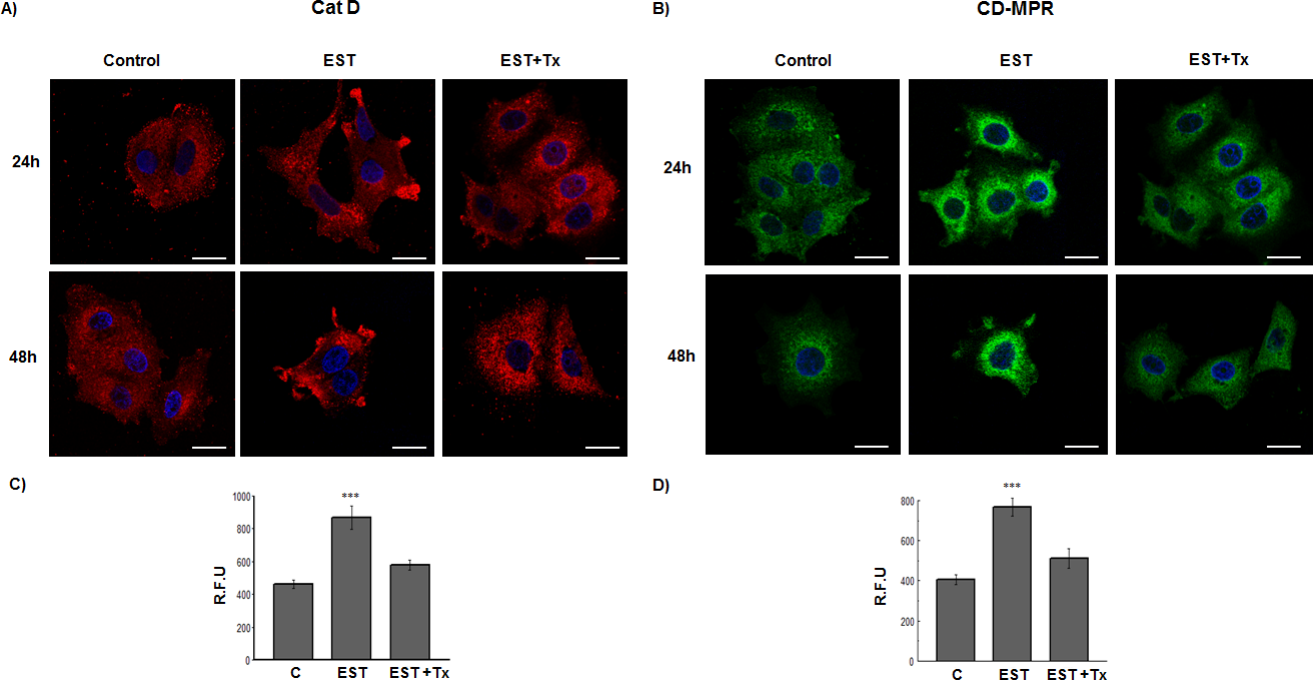
Effect of 17-β-estradiol on CatD and CD-MPR expression and distribution in MCF-7 cells. Cells were cultured for 24 or 48 h in the absence or in the presence of 20 nM estradiol (EST) or 20 nM EST with 2 μM tamoxifen (EST+Tx). Representative distribution patterns of CatD (A) and CD-MPR (B) and the fluorescence intensity quantitation, (C) and (D), respectively. Values are expressed as the means of relative fluorescence units (R.F.U.) ± SEM from 60 cells counted at each condition. (***) significant difference (p<0.001). Scale bar = 20 μm.

It was observed that 17-β-estradiol induces an increase in CatD and CD-MPR expression (Fig 6A and 6B) at 12, 24 and 48 h of incubation with the hormone, and this effect was blocked by the antiestrogen drug tamoxifen. The increment of both proteins was also observed by IFI (Fig 7B and 7D). In addition, 17-β-estradiol induced a redistribution of CatD and CD-MPR to the cell periphery (Fig 7A and 7C, respectively). The latter effect was also blocked by tamoxifen.

**Fig 8:**
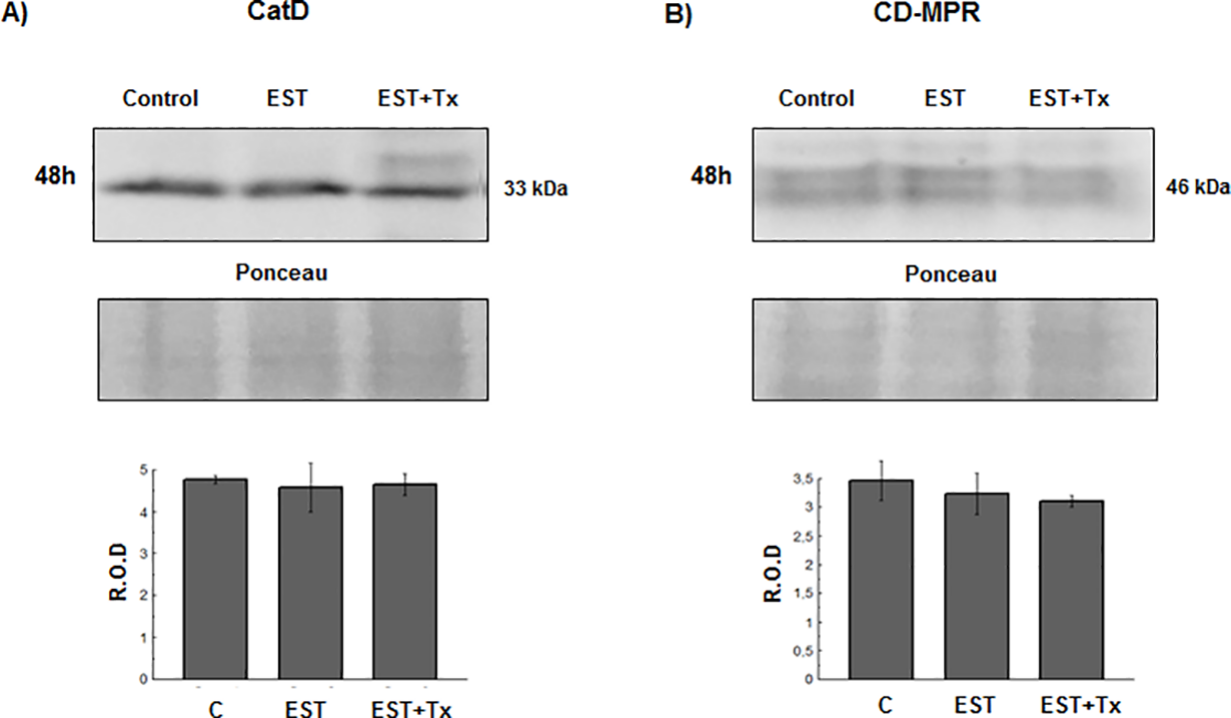
CatD and CD-MPR expression in MCF-10A human breast cells after treatment with 17-β-estradiol. MCF-10A cells were cultured for 48 h in the absence (control) or in the presence of 20 nM estradiol (EST) or 20 nM EST with 2 μM tamoxifen (EST+Tx). Representative immunoblots of CatD (A) and CD-MPR (B) from cell lysates and the corresponding band intensity quantitation by densitometry. Bars represent the means of relative optical densities (R.O.D.) from two independent experiments ± SEM. Ponceau staining was used as loading control.

A similar experiment was carried out with the non-tumorigenic MCF-10A cells, which lack the ERα. No significant changes in CatD and CD-MPR expression were observed after treatment with the hormone (Fig 8). These findings support the idea that the changes observed in MCF-7 cells were mediated by ERα.

### Estradiol induces the redistribution of CatD and CD-MPR towards common compartments

**Fig 9:**
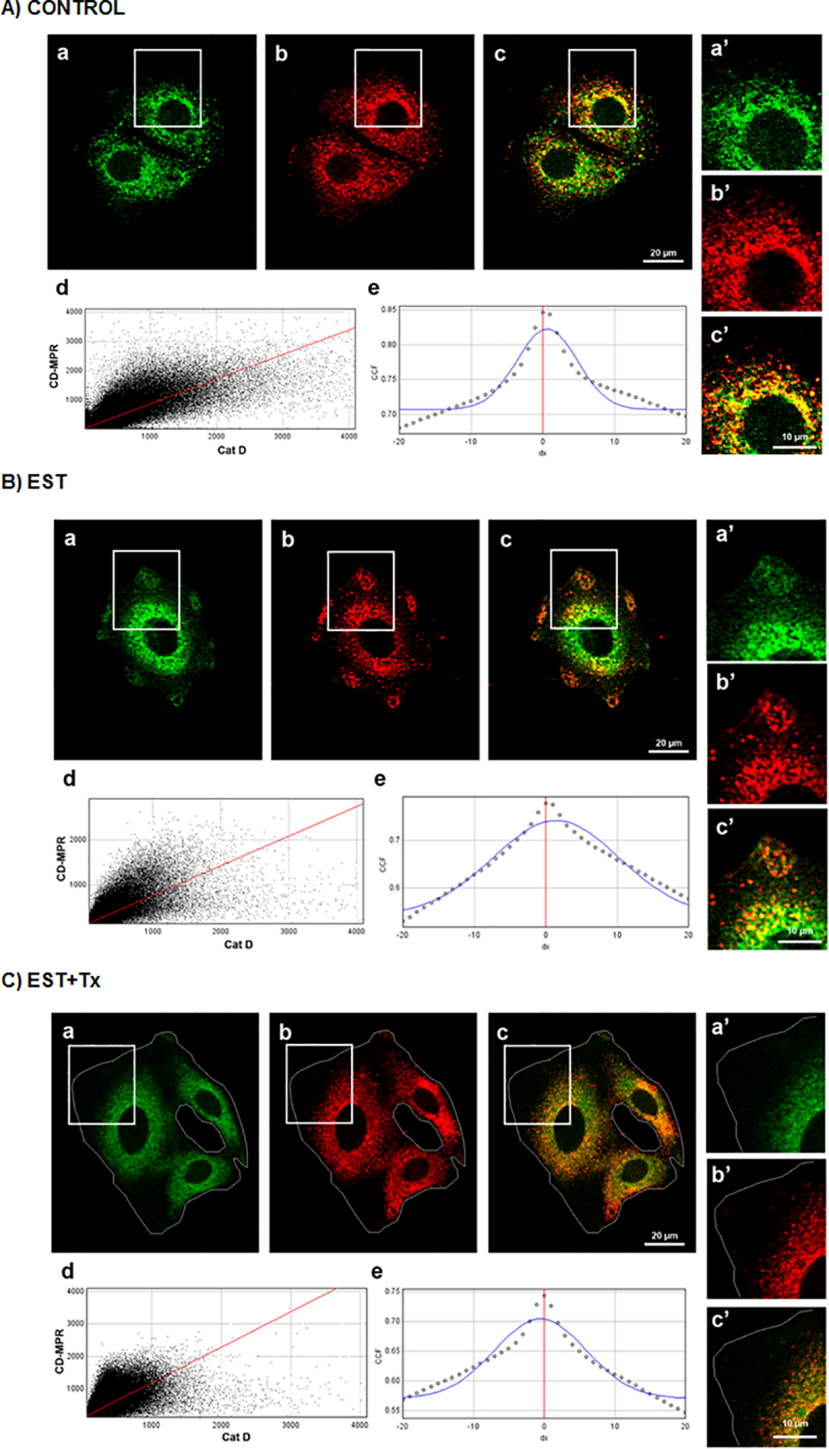
Co-localization analysis between CatD and CD-MPR in MCF-7 breast cancer cells. Representative IFI for the detection of CD-MPR (a) and CatD (b) in MCF-7 cells, control (A), treated with 20 nM EST (B) or treated with 20 nM EST+ 2 μM Tx (C). (c) Merged Images; (d) Scatter plot of co-localization events for CD-MPR and CatD; and (e) Cross-correlation function: PCCs *vs*. pixel shift (δx) for each case. (a´b´c´) amplifications of the areas limited by rectangles. PCC values: controls = 0.708 ± 0.04, EST = 0.776 ± 0.03, EST+Tx = 0.792 ± 0.05. Images are representative of three independent experiments.

By IFI, we observed that 80% of CatD co-localizes with CD-MPR (MCC-M1: 0.81 ± 0.06) and that 95% of the CD-MPR co-localizes with CatD (MCC-M2: 0.95 ± 0.05) in control MCF-7 cells. After treatment with estradiol for 24 h, both proteins redistributed partially from a mostly perinuclear location to a more peripheral granular location, maintaining the high co-localization rates (Fig 9B). This result suggests that both proteins could share intracellular compartments, either as free or complexed forms, and that they relocate together under estradiol stimulation. This effect was blocked by tamoxifen (Fig 9B).

**Fig 10:**
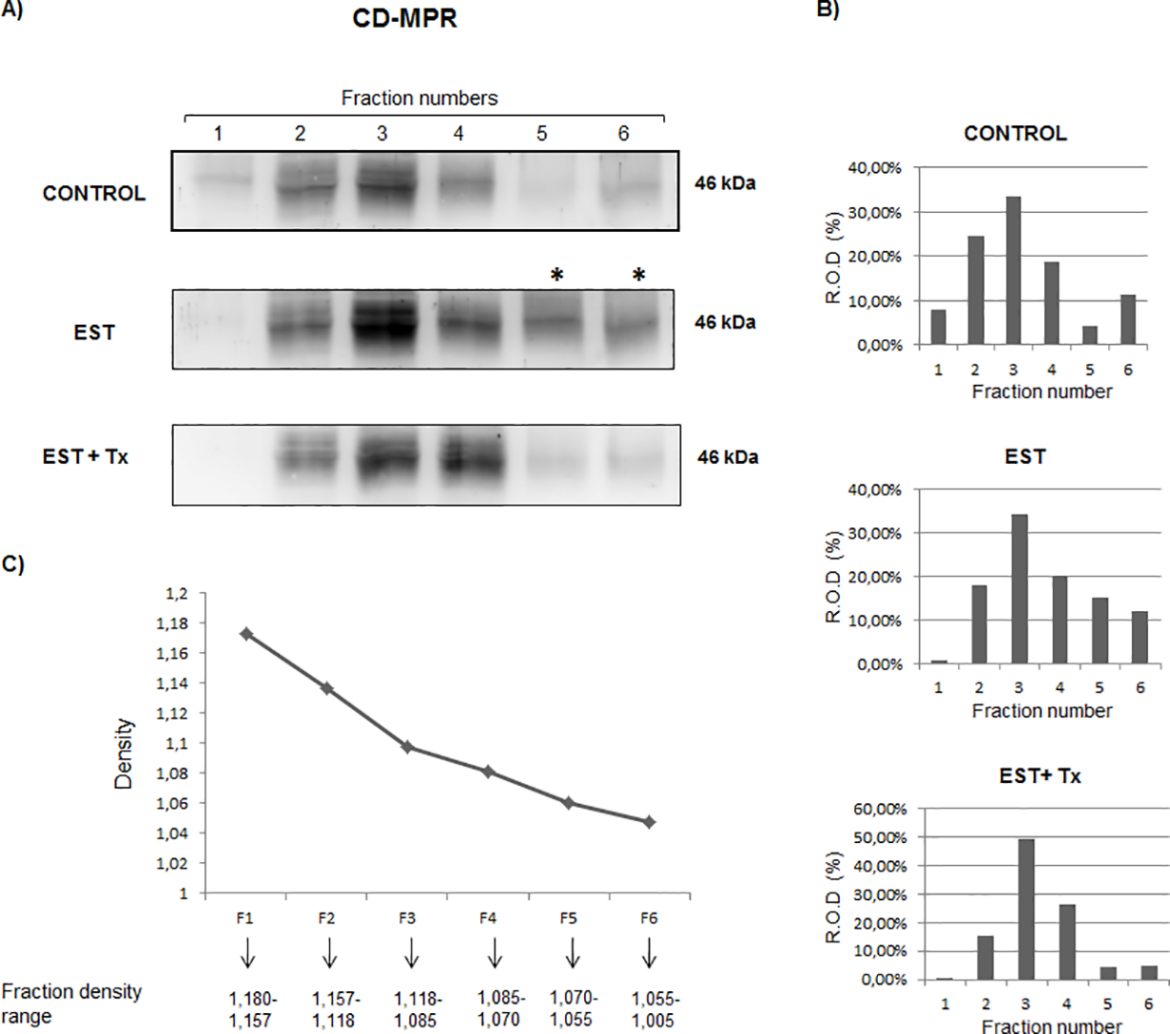
Effect of 17-β-estradiol on the distribution of CD-MPR in MCF-7 breast cancer cells. Cells were cultured in the presence of 20 nM estradiol (EST) or 20 nM EST with 2 μM tamoxifen (EST+Tx) for 24 h, and subsequently, cell lysates were loaded on sucrose gradients. Gradient fractions were analyzed by immunoblotting. (A) Detection of CD-MPR. (B) Percentage distribution quantitation obtained by densitometric analysis of each fraction. (C) Fraction densities estimated from weight/volume ratios.

To further confirm that the re-distribution of CD-MPR to the plasma membrane was driven by estradiol, we performed a subcellular fractionation by a discontinuous sucrose gradient (Fig 10). We observed that estradiol induced the appearance of CD-MPR in low density fractions enriched in plasma membrane markers [29]. Again, the stimulation with estradiol induced an increase in the CD-MPR expression. Both effects were also blocked by tamoxifen.

## Discussion

In cancer cells, CatD trafficking is altered, and this phenomenon leads to an increase in the pro-enzyme secretion [30, 31, 32]. Several mechanisms can be proposed to explain the magnitude of such secretion; e.g., the excessive expression would cause the enzyme to be secreted to the medium, a detour from the route to lysosomes or a selective transport to the extracellular medium, among others. In most cell types, acid hydrolases, including CatD, are delivered to lysosomes by mannose-6-phosphate receptors (MPRs). The present study shows that MCF-7 cells have higher levels of CD-MPR than other breast-derived cell lines (MCF-10A and MDA-MB-231), while showing the lowest levels of CI-MPR. This phenomenon would indicate that both receptors are alternative for the trafficking of mannose-6-phosphate bearing enzymes [33], and that, in these cell lines, their expression is cross-regulated. Although this hypothesis is in agreement with that proposed by other authors, it does not fully explain the fact that both receptors recognize different enzyme sets [34]. Even though in this study we do not present direct supporting evidence, the increased simultaneous expression of CD-MPR and CatD in MCF-7 cells would suggest that CD-MPR is a receptor for this enzyme. However, the role of sortilin in cathepsin D transport in breast cancer cells should not be ruled out, since this receptor in known to participate in the sorting of lysosomal proteins including cathepsin D in other cell types [12, 13]. It has been documented that sortilin can mediate cathepsin D delivery to lysosomes when the MPR pathway is impaired [12]. Exploring the role of sortilin in intracellular transport in breast cancer cells could prove to be of substantial interest for future studies.

By IFI, we observed that CatD is mostly located perinuclearly, and also dispersed in the cytoplasm. However, it is noteworthy that the enzyme is also located at the periphery of the tumorigenic (MCF-7), but not in the non-tumorigenic cells. In tumorigenic cells, the presence of CatD in the cell periphery could be attributed to vesicle accumulation for eventual secretion. In fact, about 36% of the total intracellular CatD is present in LAMP1-negative compartments in MCF-7 cells.

Apart from the differences observed in the expression of the MPRs, we have also observed that both, CD-MPR and CI-MPR, are distributed differently in MCF-7 cells; while CD-MPR is mostly concentrated in the Golgi stacks and likely in endosomal compartments, the CI-MPR is more dispersed throughout the cytoplasm and also at the cell periphery. In addition, the high degree of co-localization coefficient of the CD-MPR with golgin-97 confirms that this receptor is mostly located in the Golgi stacks, as compared to the CI-MPR. The atypical golgin-97 dispersed distribution in MCF-7 cells was consistent with the presence of a fragmented Golgi apparatus already described in these cells [35]. The perinuclear CD-MPR distribution is common in most mammalian cell types [36], while the additional dispersed signal appears to be a feature of tumorigenic MCF-7 cells. The different distribution of the two MPRs in MCF-7 cells is consistent with the idea that both proteins are not redundant but complementary [26]. On the other hand, the CI-MPR has been proposed as a tumor suppressor molecule whose expression is downregulated by estradiol [21, 37, 38]. Unlike CI-MPR, in this work we have observed that the expression of CD-MPR is significantly increased by the action of estrogen in MCF-7 cells, and that this effect is blocked by the antiestrogenic drug tamoxifen. Moreover, this effect was accompanied by an increase of CatD levels. It is noteworthy that this is the first study suggesting an estradiol-driven regulation of CD-MPR. The fact that estradiol did not induce changes in MCF-10A cells indicates that the hormonal effect on MCF-7 cells is mediated by ERα. Moreover, this hypothesis is supported by the blocking effect of tamoxifen observed in MCF-7 cells. The results obtained in other models indicate that CD-MPR expression and distribution can be modulated by testosterone [39, 40]. Whether the high levels of CD-MPR found upon estradiol stimulation are due to an increased synthesis or a diminished degradation of the receptor remains to be determined. Since the CD-MPR-encoding gene does not have hormone responsive elements, it may be possible that the levels of this receptor were regulated indirectly through the CatD response to the hormone. In fact, it has been reported that CatD can localize to the nucleus of cancer cells, where it could participate in transcription regulation by cleaving and/or interacting with nuclear proteins, thus modulating their activity [41, 42]. As in other physiological models [26], the possibility of a cross-regulation of the expression of CD-MPR and CI-MPR cannot be ruled out.

Taking into account the results obtained herein, it is of interest to elucidate the role of CD-MPR in tumor cells, and whether there exists a direct link between CD-MPR and CatD. Although CD-MPR has scarcely been explored in cancer models, some authors have proposed a pro-tumorigenic role for this receptor [43]. It is hypothesized that the CD-MPR is a receptor for CatD, which participates in the enzyme secretion by cancer cells. In this regard, we observed high co-localization levels of the two proteins, both in the perinuclear region and adjacent to the plasma membrane. In addition, estradiol induced a redistribution of both proteins to the adjacency of the cell membrane, maintaining that high co-localization. The redistribution of the enzyme induced by the hormone is consistent with the estradiol-induced-secretion reported in the literature [6, 2]. Moreover, we confirmed the estradiol-induced CD-MPR redistribution when the receptor localized to low density fractions of the discontinuous sucrose gradient. These observations are supported by a previous work performed in another model in which the redistribution of CD-MPR towards the plasma membrane was found to correlate with high CatD secretion rates [39]. Moreover, Chao et al. have suggested a role for CD-MPR in selective enzyme secretion [20].

To sum up, we suggest that the CD-MPR would be selectively re-routed together with mannose-6-phosphate ligands (e.g. cathepsins) towards the plasma membrane by some mechanism that involves recognition signals in the CD-MPR cytoplasmic domain, which are different from those motifs that are known to participate in other intracellular transport routes [44]. Subsequently, the acidic pH of the tumor microenvironment [45] would favor the ligand-receptor complex dissociation and release of the enzyme to the extracellular medium.

In conclusion, our results provide new insights to clarify the mechanism by which human breast tumor cells distribute and secrete high amounts of proteases. This process would involve a receptor-mediated selective transport regulated by estradiol. Interfering with these processes would be a new strategy for future therapies against breast cancer.

## Supporting information

S1 Data Data sheet and statistical analysis used to build graphs in Fig 1. (XLSX)

S2 Data Data sheet and statistical analysis used to build graphs in Fig 2. (XLSX)

S3 Data Data sheet and statistical analysis used to build graph in Fig 3. (XLSX)

S4 Data Data sheet and statistical analysis used to build graph in Fig 4. (XLSX)

S5 Data Data sheet and statistical analysis used to build graphs in Fig 6. (XLSX)

S6 Data Data sheet and statistical analysis used to build graphs in Fig 7. (XLSX)

S7 Data Data sheet used to build graphs in Fig 8. (XLSX)

S8 Data Data sheet and statistical analysis of Fig 9. (XLSX)

S9 Data Data sheet used to build graphs in Fig 10. (XLSX)

S1 Fig Supporting images for Fig 1. (A) and (B) Representative immunoblottings of cathepsin D with their respective loading controls. The fourth line in (B) shows MCF-7 proteins loaded at lesser concentration. (C) and (D) Representative immunoblotting of CD-MPR with their respective loading controls. Liver proteins were used as detection control for CD-MPR. (E) Representative immunoblotting of CI-MPR with its respective loading control. (B), (D) and (E) show the molecular size markers (GeneDirex Cat. PM005-0500S and Cat. PM008-0500S).
(TIF)

S2 Fig Supporting images for Fig 6. (A) Representative immunoblotting of cathepsin D with its respective loading control and the membrane showing nonspecific secondary antibody binding. (B) Representative immunoblotting of CD-MPR with its respective loading control and the membrane showing nonspecific secondary antibody binding. Alb Biot: Biotinylated bovine serum albumin used as detection control.
(TIF)

S3 Fig Supporting images for Fig 8. Immunoblottings of cathepsin D and CD-MPR with respective loading control showing the molecular size marker.
(TIF)

S4 Fig Supporting images for Fig 10. Immunoblottings of CD-MPR from the sucrose gradient fractions.
(TIF)
