# Supplementary figures and images for: Cation-dependent mannose-6-phosphate receptor expression and distribution are influenced by estradiol in MCF-7 breast cancer cells
Source: PLoS One. 2018 Aug 7;13(8):e0201844. doi: 10.1371/journal.pone.0201844 (PMC6080777; doi:10.1371/journal.pone.0201844)

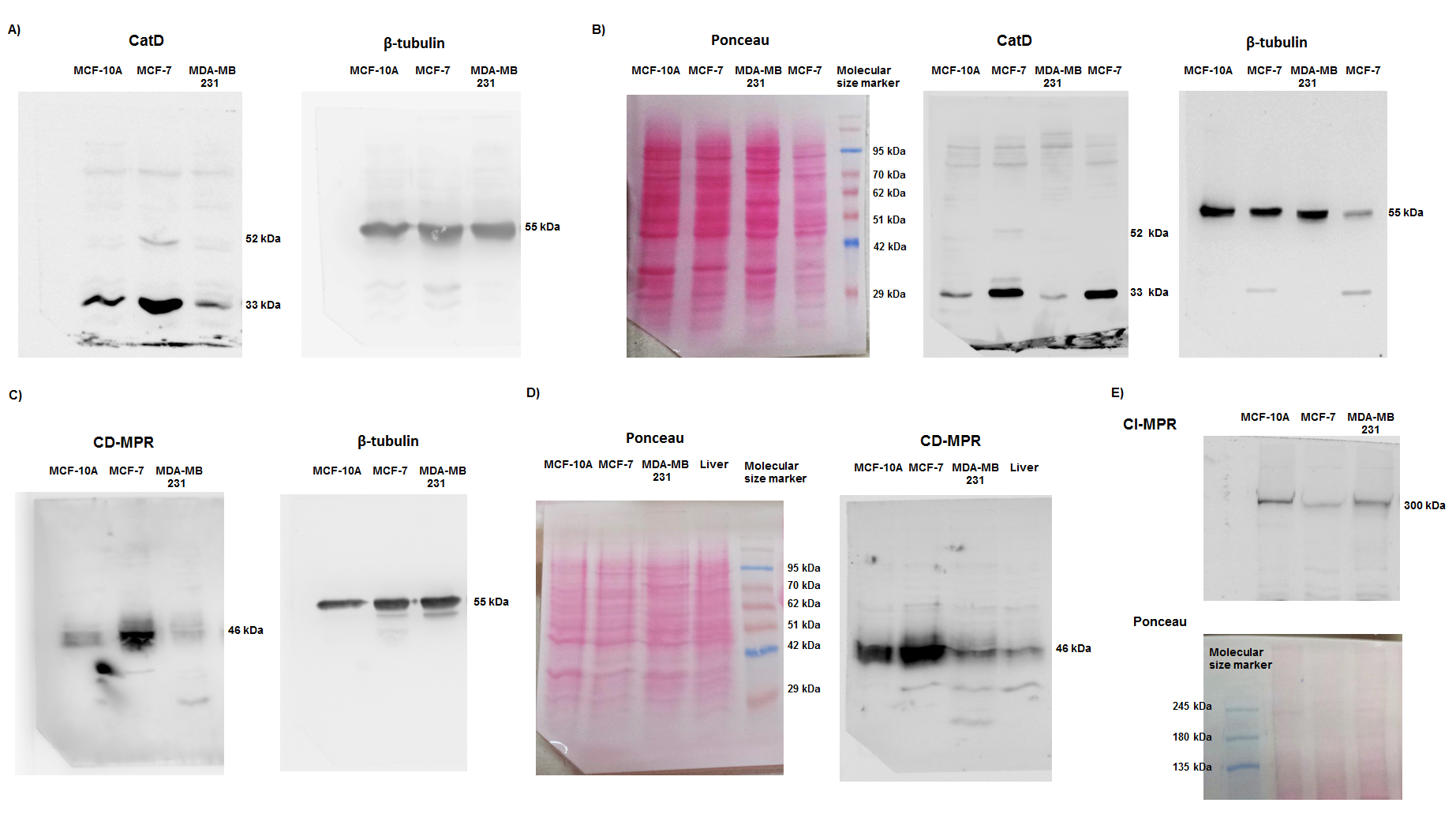

Supplement: S1 Fig — (A) and (B) Representative immunoblottings of cathepsin D with their respective loading controls. The fourth line in (B) shows MCF-7 proteins loaded at lesser concentration. (C) and (D) Representative immunoblotting of CD-MPR with their respective loading controls. Liver proteins were used as detection control for CD-MPR. (E) Representative immunoblotting of CI-MPR with its respective loading control. (B), (D) and (E) show the molecular size markers (GeneDirex Cat. PM005-0500S and Cat. PM008-0500S). (TIF) [file pone.0201844.s010.tif]

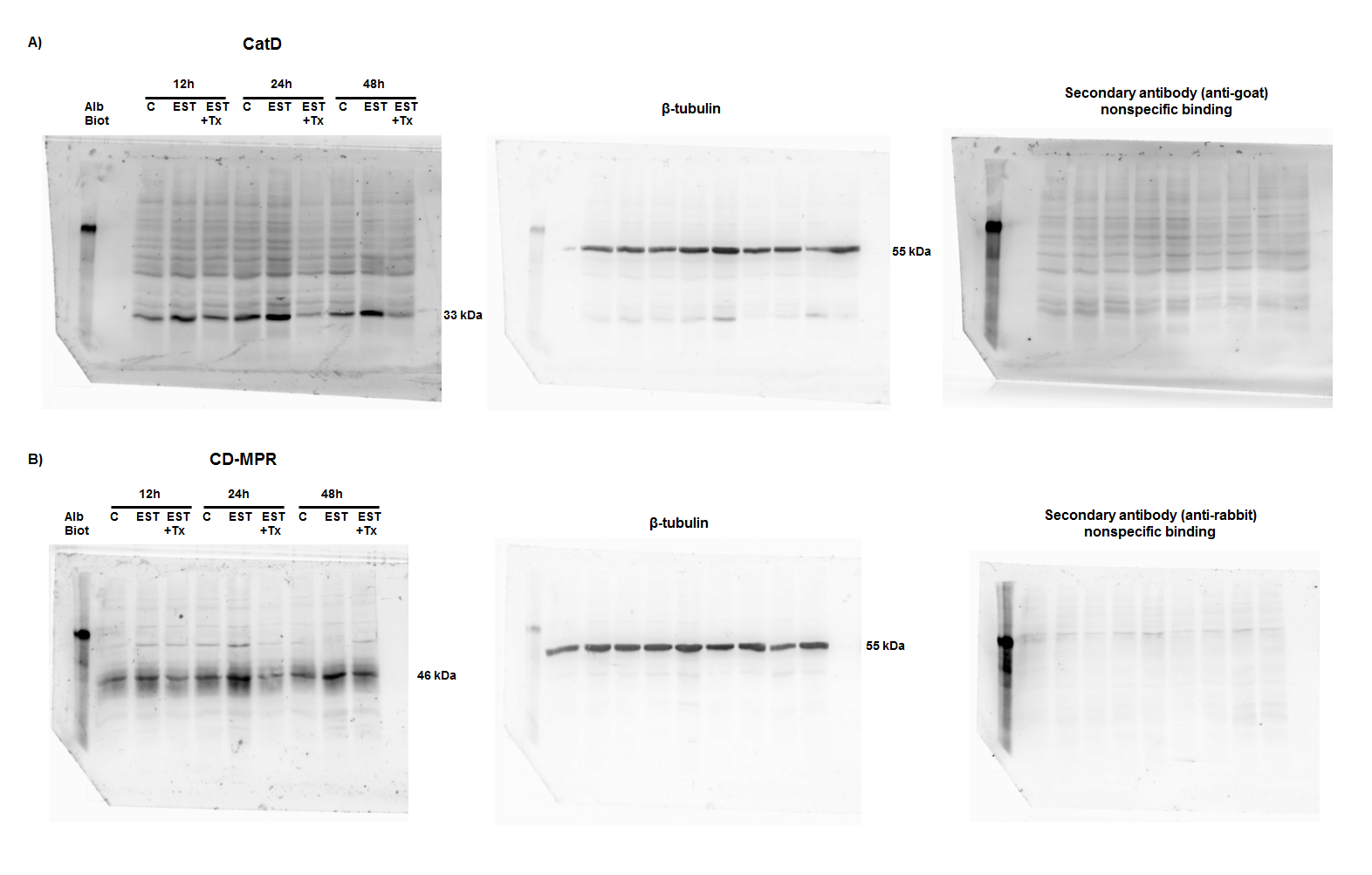

Supplement: S2 Fig — (A) Representative immunoblotting of cathepsin D with its respective loading control and the membrane showing nonspecific secondary antibody binding. (B) Representative immunoblotting of CD-MPR with its respective loading control and the membrane showing nonspecific secondary antibody binding. Alb Biot: Biotinylated bovine serum albumin used as detection control. (TIF) [file pone.0201844.s011.tif]

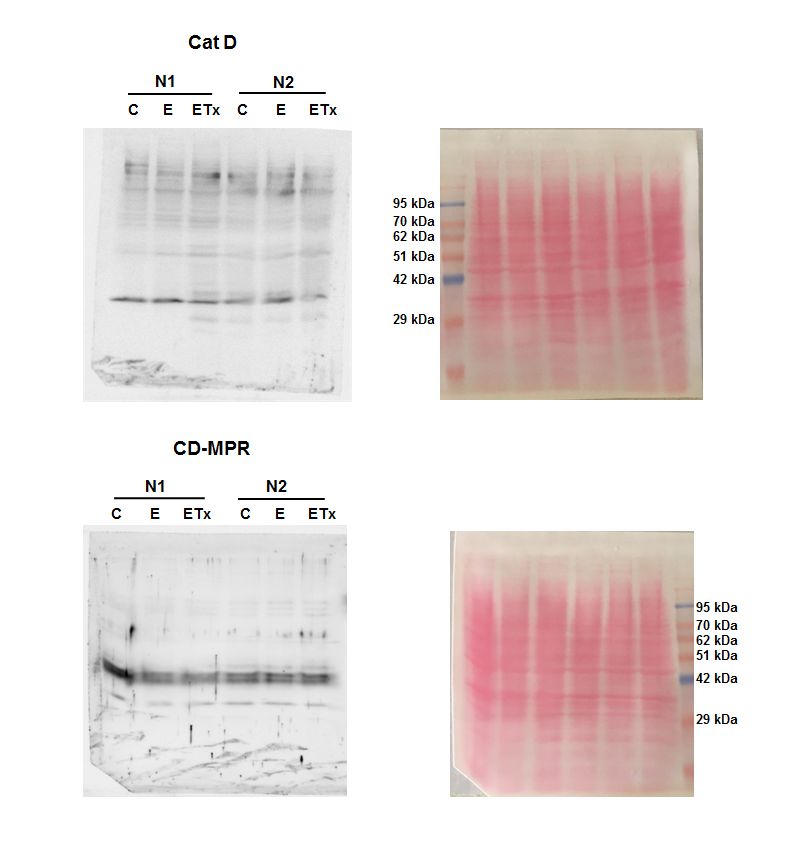

Supplement: S3 Fig — Immunoblottings of cathepsin D and CD-MPR with respective loading control showing the molecular size marker. (TIF) [file pone.0201844.s012.tif]

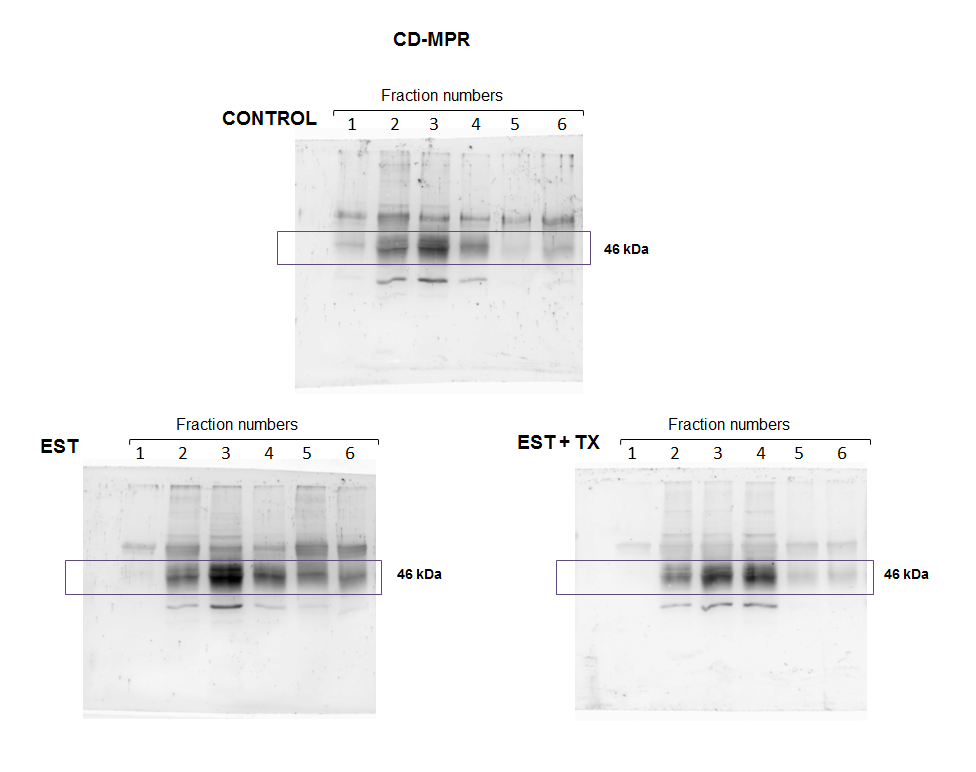

Supplement: S4 Fig — Immunoblottings of CD-MPR from the sucrose gradient fractions. (TIF) [file pone.0201844.s013.tif]
